# The merit of rural point-of-care ultrasound: Carotid pseudoaneurysm case report

**DOI:** 10.4102/phcfm.v17i1.4959

**Published:** 2025-07-23

**Authors:** Jan C. Thirion, Daniël J. Van Hoving

**Affiliations:** 1 Rural Health Services, Western Cape Government, Worcester, South Africa; 2 Division of Emergency Medicine, Faculty of Medicine and Health Sciences, Stellenbosch University, Parow, South Africa

**Keywords:** PoCUS, ultrasound, pseudoaneurysm, carotid, primary care, imaging, GP, clinic, rural

## Abstract

**Introduction:**

Extracranial carotid artery aneurysms and pseudoaneurysms are rare, comprising less than 4% of all peripheral artery aneurysms. Rural primary health care facilities often face significant challenges because of limited access to formal imaging. Point-of-care ultrasound (PoCUS) has the potential to bridge this gap, accelerating timely diagnosis and management in remote settings.

**Patient presentation:**

A 19-year-old male presented to a rural primary health care clinic in the Western Cape of South Africa with a 3-week history of left-sided neck swelling and recent odynophagia. Physical examination revealed a firm, pulsatile mass with an audible bruit.

**Management and outcome:**

Formal imaging was unavailable for several months, delaying surgical advice. However, the clinic’s newly procured mobile ultrasound allowed for PoCUS, which identified a pulsatile vascular lesion consistent with a carotid pseudoaneurysm. Computed tomography angiography confirmed the diagnosis, and the patient was referred for tertiary care where the lesion was repaired. He had vasculitis on histology and exhibited inconclusive features of a connective tissue disorder, but a definitive cause was not found. Despite multiple attempts, he could not be contacted for follow-up.

**Conclusion:**

This case highlights how PoCUS can accelerate definitive management in resource-limited settings.

**Contribution:**

Point-of-care ultrasound is potentially an effective, cost-efficient diagnostic tool in rural healthcare settings but requires significant investment in equipment and training. Further research is needed to evaluate its feasibility in South African rural health systems.

## Introduction

### Background: Primary health care and imaging access

We report a case of a young man who received prompt diagnosis and management for a carotid pseudoaneurysm because of point-of-care ultrasound (PoCUS) capability at his local primary health care clinic.

Extracranial carotid artery aneurysms and pseudoaneurysms are rare and make up less than 4% of all peripheral artery aneurysms.^1^ Causes of pseudoaneurysm commonly include infection, injury, tumour invasion and connective tissue disorders.^2,3^

This case was initially managed at a rural primary health care clinic in the Western Cape of South Africa. The clinic serves approximately 5000 patients per month with a staff complement of 12 nurses, one medical officer and an intern doctor. HIV, lifestyle diseases and tuberculosis are the predominant chronic diseases seen there.

Emergency cases are assessed and stabilised before referral to the secondary-level hospital situated 30 km away.

All patients are referred for basic radiology investigations. The waiting period is seven days for plain radiographs and in excess of three months for ultrasound examinations. Computed tomography (CT) is a limited resource that can only be ordered by in-hospital physicians. The secondary hospital covers a 40 129 km^2^ drainage area, serving a population of 781 304 (2022 census).^4^ The hospital employs one part-time specialist radiologist and two sonographers.

### Ethical considerations

Ethical clearance to conduct this study was obtained from the Stellenbosch University Health Research Ethics Committee (No. C24/12/034). The patient consented verbally but was later not reachable for written consent.

## Patient presentation: A young man with neck swelling

A 19-year-old male presented to the clinic with a three-week history of a left-sided neck swelling, accompanied by odynophagia for the previous two days. He reported no preceding trauma, chronic illnesses, previous surgeries or notable family history. His only prior contact with the clinic was two months earlier when he received empiric antibiotic treatment for a sexually transmitted infection (STI). Sexually transmitted infections are treated without bacterial confirmation in this setting as per local protocols.

His vital signs were within normal limits. Systemic examination revealed a unilateral, left-sided, firm and pulsatile mass at the base of his neck. It was approximately 6 cm × 4 cm in size with an audible bruit. No lymphadenopathy was present. Further cardiovascular examination was normal. Other systemic examinations were unremarkable. He exhibited no signs of trauma or infection and appeared clinically well. Rapid tests for HIV and syphilis (done as per local protocol) were negative at the time; further blood tests were deferred to the secondary level.

### Diagnosis, referral and management

The case was discussed with the surgery team at the secondary hospital. A vascular lesion was deemed unlikely, and they suspected the mass was a lymph node with transmitted pulsation from an underlying vessel. Admission for emergency imaging was denied, and elective ultrasound was advised.

The next available formal ultrasound appointment was five months away because of system-related resource constraints. However, a mobile ultrasound machine was procured in this health subdistrict six months prior, for use at the clinic level. Arrangements were made by the primary clinician to bring the machine to the clinic the next day.

**FIGURE 1:**
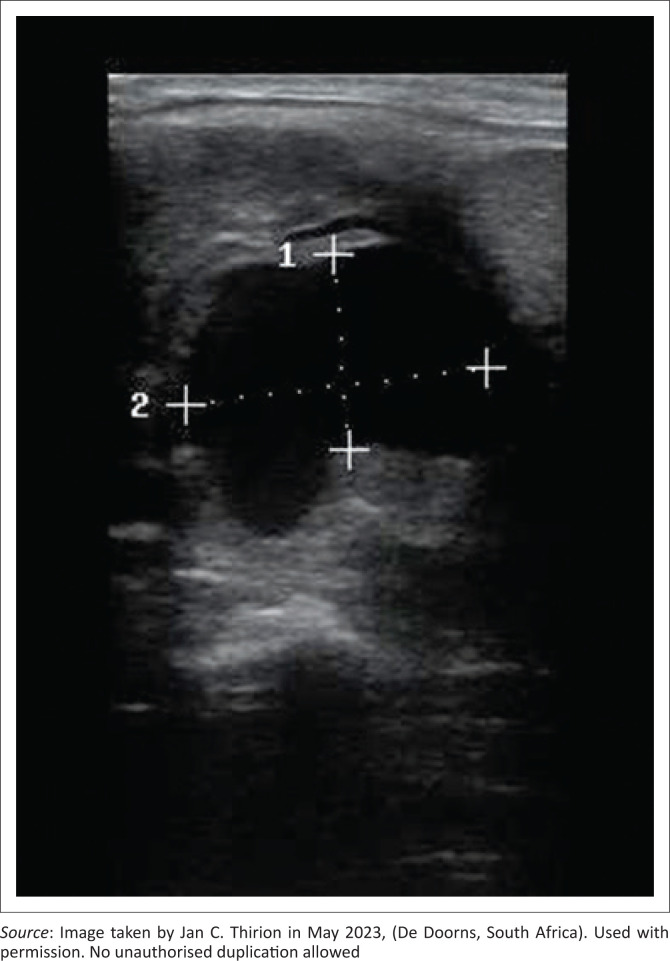
Image of pseudo-aneurysm taken during point-of-care ultrasound examination.

The primary health care clinician performed a PoCUS examination, which revealed a large pulsating vascular lesion communicating with the bifurcation of the left carotid artery (Figure 1). A carotid pseudoaneurysm was suspected.

The patient was re-discussed with the secondary hospital and was promptly accepted for an urgent CT angiogram, which confirmed the diagnosis of a carotid pseudoaneurysm. Admission bloods were normal; creatinine 75 umol/L, white cell count 7.23 × 10^9^/L, haemoglobin 13.9 g/dL and platelet count 225 × 10^9^/L. The patient was transferred for tertiary care, where the lesion was repaired using an autologous venous graft. Histological samples indicated vasculitis; a tissue sample and blood bacterial cultures were negative. Unfortunately, the tissue graft failed within a few months (again recognised by the primary care physician), and the pseudoaneurysm was subsequently successfully repaired using a polytetrafluoroethylene (PTFE) graft.

## Management and outcome

### Recovery, follow-up and aetiological considerations

Six months after the second surgery, the patient was doing well and had experienced no ill effects of the original pathology.

He was evaluated for a connective tissue disorder but did not fully meet the criteria for Marfan syndrome, although Ehlers-Danlos syndrome remained an unexplored possibility.

He did not attend subsequent appointments, including an appointment for genetic screening, after relocating to pursue his tertiary education. Despite several attempts, contact with the patient could not be re-established.

The exact cause of the pseudoaneurysm could not be established. The potential existence of a connective tissue disorder or links to the previously suspected STI could not be confirmed.

## Discussion

### The role and limitations of point-of-care ultrasound in primary health care

Primary health care PoCUS capability improved the management of this case by expediting admission and avoiding potentially dangerous delays. This reduction in time-to-diagnosis is a known benefit of PoCUS, as demonstrated in emergency centres.^5^

South Africa faces inequitable distribution of formal imaging services in its government facilities,^6^ leading to delayed care for rural patients, and PoCUS has the potential to improve access to imaging by empowering rural healthcare providers.^7^ For conditions suspected clinically that can reliably be ruled out using PoCUS, such as abdominal aorta aneurysms, deep vein thromboses and pleural effusions, pressure on hospital emergency centres and rural transport resources may be reduced by shifting diagnostic tasks to the primary health level.^8^ Point-of-care ultrasound has also been shown to improve patient satisfaction in hospital settings in the United States^9^ as well as family physician job satisfaction in Canada.^7^

However, PoCUS is not without its own drawbacks. Widespread adoption of PoCUS would be an inherently expensive undertaking because of the costs of equipment and additional training required for primary health care doctors. This effort can be justified, given the benefits and cost-effectiveness PoCUS has shown in other health systems.^10,11^ Further pitfalls include the potential for misdiagnosis because of incorrect interpretation of PoCUS findings,^11^ difficulties fitting PoCUS into clinical consultations because of time constraints^7^ and the possibility of false patient re-assurance or conversely, heightened patient anxiety because of the addition of PoCUS during consultations.^9^

More research in the context of primary health care in South Africa is needed to determine whether expanding rural PoCUS capability should be prioritised.
